# Essential Oils: Chemistry and Mechanisms of Anticonvulsant Action

**DOI:** 10.3390/ijms27114691

**Published:** 2026-05-22

**Authors:** Lígia Salgueiro, Mónica Zuzarte, Jeremias Justo Emídio, Diogo Vilar da Fonsêca, Damião Pergentino de Sousa

**Affiliations:** 1 Faculty of Pharmacy, University of Coimbra, Azinhaga de S. Comba, 3000-548 Coimbra, Portugal; ligia@ff.uc.pt (L.S.); mzuzarte@uc.pt (M.Z.); 2 Chemical Engineering and Renewable Resources for Sustainability (CERES), Department of Chemical Engineering, University of Coimbra, 3030-790 Coimbra, Portugal; 3 Center for Innovative Biomedicine and Biotechnology (CIBB), Coimbra Institute for Clinical and Biomedical Research (iCBR), Faculty of Medicine, University of Coimbra, Azinhaga de S. Comba, 3000-548 Coimbra, Portugal; 4 Clinical Academic Centre of Coimbra (CACC), 3004-504 Coimbra, Portugal; 5 Pharmaceutical Chemistry Laboratory, Department of Pharmaceutical Sciences, Federal University of Paraíba, João Pessoa 58051-900, Brazil; jeremiasjusto@gmail.com; 6 Academic Health Unit, Federal University of Campina Grande, Cuité Campus, Campina Grande 58175-000, Brazil; divilar@hotmail.com

**Keywords:** adulteration, epilepsy, convulsion, natural products, metabolites, medicinal plants, essential oil, volatiles, pentylenetetrazole, terpene

## Abstract

Essential oils have attracted increasing attention due to their bioactive properties. This review focuses on their anticonvulsant potential by exploring the relation between the chemical composition of essential oils and the mechanism of action underlying this effect. Evidence from in vivo and ex vivo studies is presented to identify structure–activity relations and to distinguish well-supported effects from preliminary findings. Moreover, essential oil’s quality is vital to ensure safety and efficacy in pharmacotherapeutic approaches. For this reason, factors including extraction and analytical methods as well as authenticity assessment are discussed due to their impact on pharmacological consistency and reproducibility. Overall, this review highlights key compounds and mechanisms contributing to anticonvulsant activity, identifies current limitations in the literature, and outlines priorities for future research aimed at validating essential oils as potential complementary therapeutic agents in seizure management.

## 1. Introduction

Essential oils (EOs) are complex mixtures of volatile compounds derived from natural raw materials of plant origin with a wide range of applications due to their important biological properties [1,2]. Their chemical composition, predominantly consisting of monoterpenes, sesquiterpenes, and phenylpropanoids, may vary significantly depending on genetic, environmental, and methodological factors [3,4]. In this context, the appropriate selection of extraction methods, combined with thorough chemical characterization is essential to ensure quality, authenticity, and reproducibility [5,6,7]. Analytical standardization is, therefore, particularly relevant, given the intrinsic natural variability that may compromise the biological activity and consistency of EOs [8,9,10,11].

**Figure 1:**
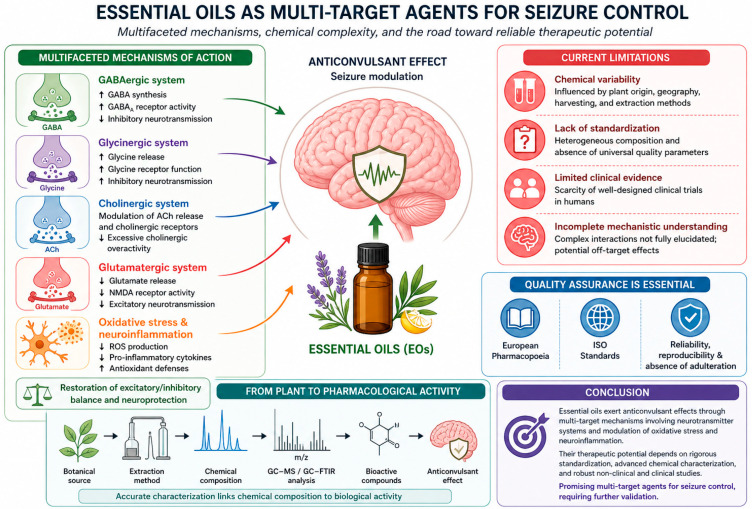
Main anticonvulsant mechanisms of action, quality assurance, and limitations in pharmacological studies of essential oils.

Advances in research have highlighted the therapeutic potential of EOs. These complex mixtures of bioactive compounds act on various biological targets, leading to a wide range of pharmacological effects, including antiviral, anti-inflammatory, antimicrobial, and effects on the central nervous system [12,13,14]. In fact, their psychopharmacological properties have been extensively studied, such as their anticonvulsant activity in both in vivo and ex vivo models. These effects are mainly attributed to the modulation of neurotransmitter systems including the GABAergic and glutamatergic pathways, as well as mechanisms related to oxidative stress and neuroinflammation [15,16]. Considering the therapeutic limitations of currently available antiepileptic drugs, the integration of robust chemical data with mechanistic studies is essential to better elucidate the potential of EOs as promising sources of anticonvulsant agents [17,18]. Accordingly, this review examines extraction and analytical methods for EOs, as well as their main anticonvulsant mechanisms of action, highlighting the importance of quality control of these products and their potential use as drugs for the treatment of epilepsy. Figure 1 summarizes the anticonvulsant mechanisms of action and chemical aspects that may influence pharmacological activity.

## 2. Materials and Methods

The search was performed on PubMed/MEDLINE. The following terms were used in combination within search strings: “anticonvulsant”; “epilepsy”; “essential oils”; “analysis”; “quality control”; “extraction”. The search was restricted to English and experimental studies.

## 3. Essential Oils Quality

### 3.1. Essential Oils Extraction

Essential oils are highly valuable natural products due to their wide range of biological activities, including antimicrobial, antioxidant, anti-inflammatory, and neuroactive effects. These properties underpin their growing relevance in pharmaceutical, food, agricultural, and cosmetic applications. In addition to their bioactivity, their distinctive fragrances (e.g., citrus oils) contribute to their use in perfumery and as flavour and aroma enhancers. EOs also serve as precursors for the synthesis of other compounds, such as turpentine oil [19,20].

Numerous factors can influence the quality of EOs, including the extraction process itself, which is an essential element in improving the overall quality of these oils. According to ISO 9235 [21], established by the International Organization for Standardization on EOs [22], EOs are defined as products obtained through steam distillation, mechanical processes applied to the epicarp of citrus fruits, or dry distillation. Among these, steam distillation remains the most widely used method, reflecting its effectiveness in preserving the aromatic and functional properties of plant constituents. Depending on the system, it may be performed as hydrodistillation, where water is added to the still, or as direct steam distillation, without the addition of water. Modern steam distillation systems consist of a biomass container, condenser, oil separator, and steam generator. Perforated plates are used to ensure efficient steam flow and prevent compaction of plant material. The condensed liquid is then separated into EO and hydrosol, typically using a Florentine flask. Distillation time varies depending on the raw material, ranging from short durations for flowers to several hours for roots or sesquiterpene-rich materials [23,24]. In contrast, dry-distillation is applied to materials such as wood, bark, roots, or gums, without the use of water or steam. This process involves heating in the absence of atmospheric oxygen, typically in a closed vessel, preventing combustion, like Birch tar essential oil.

Certain raw materials require specific extraction conditions. For instance, orris root distillation is performed under slightly elevated pressure to facilitate the extraction of high-boiling compounds such as irones. Hard materials may also require pre-treatment, such as grinding in the presence of water, to improve extraction efficiency. Additionally, mechanical stirring is often applied to enhance mixing and promote EO release [23].

Importantly, high-temperature extraction processes can also lead to the formation of artifacts—compounds that do not naturally exist in the plant. One notable example is chamazulene, a blue bicyclic sesquiterpene formed in the EO of *Matricaria recutita* during distillation [25]. Chamazulene is derived from matricin through chemical reactions, including dehydrogenation, dehydration, and ester hydrolysis. Additionally, degradation processes such as ester hydrolysis may occur during extraction, which is particularly relevant for EOs rich in esters. For instance, *Lavandula angustifolia* contains high levels of linalyl acetate, while *Elettaria cardamomum* is rich in terpinyl acetate, making these oils especially susceptible to such degradation [26]. These transformations may modify the chemical profile of the EO and, consequently, alter its biological activity, including its pharmacological potency and overall therapeutic effects.

For fruits of the *Citrus* genus, the cold-pressing method is the most suitable for EO extraction due to the presence of numerous thermolabile and unstable compounds, particularly aldehydes. *Citrus* EOs, derived from various fruits of the *Citrus* genus, are among the most widely produced on an industrial scale. Examples of citrus fruits used for the extraction of high-value EOs include *Citrus bergamia*, *C. aurantium*, *C. aurantifolia*, *C. sinensis*, and *C. reticulata* [27].

Certain EOs undergo treatments to remove specific components, resulting in products such as terpene-less or sesquiterpene-less oils, or oils corrected by the partial removal of substances like furanocoumarins in *Citrus* oils [28]. These modifications are reflected in the product names, often accompanied by descriptive adjectives such as decolorized EO, washed EO, or iron-removed EO.

In other cases, EOs may require rectification, a process involving the redistillation of crude oil to eliminate undesirable impurities. These impurities can include low-volatility compounds, such as high-molecular-weight phenols carried over during steam or water distillation. Rectification also removes highly volatile compounds with unpleasant odours that can negatively affect the oil’s top note. Additionally, rectification can enhance the concentration of desired components, such as increasing 1,8-cineole content in lower-grade eucalyptus oil [29].

In addition to EOs, the industry also uses other types of aromatic extracts obtained through various extractive processes. The ISO 9235:2013 standard provides definitions for both the natural raw materials and the derived products within the aromatic industry [30]. Some of the most commonly used extracts obtained from EOs are as follows (Table 1).

Another method gaining attention, especially for laboratory or small-scale applications, is microwave-assisted hydrodistillation. Compared to conventional hydrodistillation, this technique reduces extraction time and energy consumption, and typically yields a higher final product quantity. It is valued for its gentle heating, which minimizes thermal degradation of the plant material [31]. More recently, solvent-free microwave extraction has been proposed as an environmental friendly method to extract these aromatic extracts. This technique involves microwave-assisted dry distillation of fresh plant material without the addition of water or organic solvents. During the process, selective heating of the plant’s water content causes tissue swelling and the rupture of secretory structures. The aromatic extract is then released through azeotropic distillation, while excess water returns to the extraction vessel, restoring the plant’s original water content [32]. Another relevant technique highly used in the industry resorts to supercritical fluid extraction (SFE). This method is quite effective as it enables a rapid extraction while requiring only moderate temperatures, thus avoiding the degradation of labile compounds. Harmful solvents are also avoided, as supercritical fluids are used. These fluids have intermediate properties among liquids and gases, and their strength can be controlled by adjusting the pressure and/or temperature, which enhances the selectivity of the solvent. Carbon dioxide (CO_2_) is the preferred solvent for SFE due to its non-toxic, environmentally safe, and non-flammable nature. It is chemically inert, has low viscosity and surface tension, high diffusivity, and is cost-effective. Additionally, CO_2_ is readily available and can be easily removed from the extract after decompression, leaving no residual solvent in the final product. Moreover, CO_2_ can be recycled, reducing its environmental impact [33]. When only small amounts of plant material are available, headspace-based techniques may be employed to capture volatile compounds without requiring conventional extraction procedures [34,35]. Overall, extraction conditions play a critical role in determining the chemical composition of volatile oils, which directly influences their quality, uniformity, and biological activity.

### 3.2. Essential Oils Chemical Characterization

Essential oils are complex mixtures of volatile compounds with diverse structures and functional groups. They are mainly composed of monoterpenes, sesquiterpenes, and phenylpropanoids, including their oxygenated derivatives [36], which largely define their chemical diversity and biological properties. The composition of EOs is highly variable and depends on both extrinsic factors such as environmental and geographic conditions and intrinsic factors, including seasonal, ontogenetic, and genetic variations [37]. Among these, genetic variation plays a critical role as, when sufficiently pronounced, can lead to the emergence of distinct chemical profiles within the same species, known as chemotypes. These chemotypic variations are particularly important, as they can significantly affect the concentration of bioactive compounds and, consequently, the biological activity of EOs. For this reason, industries often select chemotypes with desirable chemical profiles in order to obtain high-quality products with consistent and effective biological properties.

Given that the pharmacological effects of EOs are strongly dependent on their chemical composition, variability represents a critical challenge for their therapeutic application. In the context of anticonvulsant activity, research on EOs and their major constituents, including monoterpenes, has demonstrated distinct neuropharmacological activities, indicating that compositional differences may modulate their overall anticonvulsant effects [16]. Therefore, rigorous chemical characterization and standardization of EOs are essential to ensure reproducibility, safety, and consistent therapeutic efficacy.

To address this variability, several organizations, including the European Pharmacopoeia and the International Standard Organization (ISO), have established analytical guidelines for EO quality control [22].

Quality assessment encompass sensory evaluation as well as physical and chemical analysis. Physical parameters include EO’s density [38], refractive index [39], and optical rotation [40], commonly used to verify purity and consistency [28], while chemical tests, including acid value [41] and peroxide value [42], provide information on degradation and oxidative status. Together, these parameters ensure compliance with safety and quality standards required for pharmaceutical and commercial applications [43].

To further assess their chemical composition, EOs are typically analyzed through gas chromatography (GC). This technique is crucial for the precise identification and detailed characterization of complex volatile substances, allowing comparisons with Pharmacopoeia monographs and ISO norms. Guidelines for GC analysis using capillary columns [44] and general guidance for interpreting chromatographic profiles [45,46] are also available. Moreover, chromatographic profiling, based on representative and characteristic compounds and their concentration ranges, is widely employed to define EO quality and authenticity, factors that are strongly associated with their biological and therapeutic efficacy. These profiles are typically established using a large number of high-quality samples collected across different production conditions and are often incorporated into monographs and ISO standards. For example, bitter fennel—*Foeniculum vulgare* subsp. *vulgare* is classified into two chemotypes (*trans*-anethole type and phellandrene type [47]), while *Matricaria recutita* (syn. *Chamomilla recutita*, *Matricaria chamomilla*) also presents two chemotypes (Egyptian type and Hungarian type, [48]), each with distinct chromatographic profiles. These standards further provide industry guidelines that ensure consistency, stability, quality, and regulatory compliance [49].

Advanced analytical techniques, including GC coupled with mass spectrometry (GC–MS) and nuclear magnetic resonance (NMR), play a crucial role in the accurate identification of EO constituents, particularly in complex mixtures. These techniques are essential not only for structural elucidation but also for the identification of compounds potentially responsible for biological activities, including anticonvulsant effects.

In gas chromatographic analyses that primarily employ a flame ionization detector (FID), retention data, specifically retention indices, serve as the principal means for peak identification. The most common methods for calculating these indices include Kováts logarithmic equation [50], for isothermal conditions, and van den Dool and Kratz non-logarithmical formula [51], for temperature-programmed conditions. Typically the analysis of EOs employs non-polar polysiloxane-based stationary phases together with moderately polar phases like polyethylene glycol. This combination helps prevent the co-elution and provides comprehensive, standardized chromatographic profiles. Because EOs are complex mixtures containing isomers and homologous groups from diverse chemical families (alcohols, aldehydes, esters, ketones, oxides, and ethers) using columns with different polarities and orthogonal selectivity improves separation and supports more accurate identification.

Hyphenating GC with spectroscopic techniques, like mass spectrometry (MS) and Fourier Transform Infrared Spectroscopy (FTIR), improves compound identification. GC-MS is widely employed to identify complex volatile compounds using specialized detectors such as mass-selective detector or ion traps.

Mass spectrometry identifies compounds by converting them into ions, and measuring their mass to charge ratios (*m*/*z*). The resulting spectrum shows fragment patterns and relative abundances, helping determine the compound’s structure and molecular mass [52]. Due to the high reproducibility of spectra obtained through electron impact at 70 eV, it is possible to compare them with spectra available in published or commercial collections [53,54].

Gas Chromatography-Fourier Transform Infrared Spectroscopy (GC-FTIR) is useful as it can distinguish between isomers, particularly sesquiterpenes that often have identical mass spectra. GC–FTIR provides reproducible spectra that can be compared with databases. Identification is improved by combining retention indices from different columns with MS and FTIR data. This integrated approach, supported by extensive RI and MS libraries, enables more complete and reliable compound analysis [55]. In addition to structural and compositional analysis, techniques like GC–isotope ratio mass spectrometry (GC–IRMS) offer complementary insights by focusing on the origin of the compounds. This method combines GC to separate individual components and isotope ratio MS to measure the ratio of stable carbon isotopes, such as ^13^C/^12^C or ^14^C/^12^C. It is often used to determine whether a substance is of natural or synthetic origin, based on its carbon isotope signature [56].

Given the strong dependence of biological activity on chemical composition, the integration of chromatographic and spectroscopic techniques is fundamental for establishing reliable chemical profiles and ensuring the reproducibility of pharmacological effects. This is particularly critical in anticonvulsant applications, where even small variations in EO composition may lead to differences in efficacy and safety outcomes [16].

Considering that EOs may contain hundreds of constituents, co-elutions are inevitable, and the limited peak capacity of a single column often restricts the complete separation of all compounds in one-dimensional GC.

To overcome these limitations, multidimensional GC (MDGC), including heart-cut (GC–GC) and comprehensive (GC × GC) approaches, has been developed to enhance selectivity, peak capacity, and resolution. GC × GC, in particular, improves compound separation and identification accuracy when coupled with spectrometric detection systems [57,58]. This is especially relevant for highly complex oils, such as vetiver EO, where conventional GC analysis is often insufficient for accurate characterization [59].

MDGC is also increasingly applied to enantioselective analysis. Chiral compounds, commonly found in EOs, can be quantified by their enantiomeric excess, which varies according to species and chemotype and is relevant for assessing purity, origin, and potential adulteration. Additionally, enantiomeric composition can influence organoleptic properties, as illustrated by limonene enantiomers, which exhibit distinct sensory characteristics [49].

Capillary GC with chiral stationary phases, such as cyclodextrin derivatives, remains the standard approach for enantiomeric analysis, as described in ISO 22972:2004 [60]. However, the limitations of one-dimensional GC have led to the increasing use of multidimensional approaches, which provide enhanced resolution and more reliable identification of chiral compounds [57].

In addition to chromatographic techniques, nuclear magnetic resonance (NMR) spectroscopy, particularly ^1^H and ^13^C NMR, is also applied in the analysis of EOs, as they provide information on the molecular structure, composition, and dynamics. Owing to its quantitative capability, NMR enables the simultaneous identification and quantification of multiple components in complex mixtures, making quantitative NMR (qNMR) a powerful analytical tool [61,62]. Applied to commercial EO samples, NMR allows the detection of vegetable oil adulterants via characteristic signals, supporting its role as a reliable technique for assessing EO purity and authenticity [61].

In addition, organizations such as the French Standards Association (AFNOR) provide complementary methodologies for assessing EO quality, including the detection of adulteration and the quantification of potentially harmful compounds. These standards contribute to ensuring the authenticity, stability, and safety of EOs, reinforcing their suitability for therapeutic use [49].

## 4. Essential Oil Adulteration

**Figure 2:**
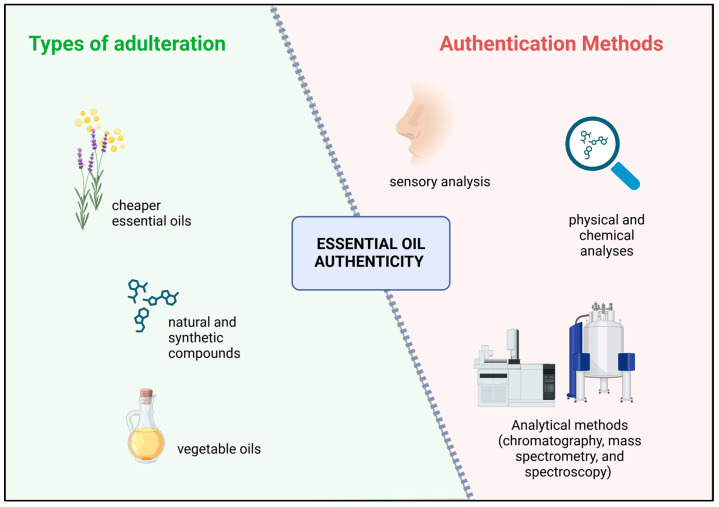
Recommended authentication tests to assess common adulterations found in EOs.

Given the strong dependence of biological activity on chemical composition, the authentication of EOs is critical to ensure both quality and pharmacological efficacy. As discussed in Section 3.2, variations in chemical composition may significantly influence the biological effects of EOs, including their anticonvulsant effects. Adulteration represents a major challenge in the EO industry and is driven by multiple factors, including economic value, aroma similarities, limited regulation in certain regions, and natural variability. Consequently, quality control is essential not only for detecting adulterations but also for ensuring the safe and effective therapeutic use of EOs. Common adulterations practices include the addition of similar or cheaper EOs, natural or synthetic compounds, and even vegetable oils, all of which may significantly alter both chemical composition and bioactivity. A summary of the main types of adulteration and detection techniques employed is summarized in Figure 2 and detailed below.

Sensory analysis involves evaluating the organoleptic properties of EOs, including characteristics such as aroma, color, texture, and taste. The results of these analyses can range from basic compliance or non-compliance with standards to more detailed assessments, such as statistical comparisons with reference EOs. In such analyses, trained professionals use their senses to identify adulterations, such as abnormal colors, textures, or odors. More recently, electronic sensors known as electronic noses (e-noses) have been used in sensory evaluation of EOs for adulteration assessment. These devices offer enhanced ease of use, high sensitivity, real-time detection, and non-destructive properties. In fact, when compared to traditional analytical methods, e-noses provide superior performance by offering quicker and more efficient adulteration detection, often with greater accuracy [63]. For example, this system has been successfully applied to identify the adulteration of *Cistus ladanifer* EO with *Pinus pinaster* EO [64]. Physical and chemical analyses provide additional layers of quality assessment. Parameters such as density, refractive index, and optical rotation are commonly used to evaluate EO purity and consistency. For example, *Citrus* EOs rich in (+)-limonene presents a lower specific gravity and optical rotation when mixed with turpentine [65]. Moreover, the addition of synthetic anethole to star anise (*Illicium verum* L.) EO results in changes in optical rotation. Although the assessment of these properties determines whether EOs meet quality standards or standardized specifications, noncompliance does not necessarily indicate adulteration. Various factors, including aging, processing, and storage conditions may also influence these values through processes such as racemization or terpene polymerization.

To detect more subtle adulterations, advanced analytical techniques are required. Chromatographic and spectroscopic methods allow detailed compositional analysis and the identification of characteristic marker compounds. For example, the evaluation of normalized peak areas and enantiomeric composition is particularly useful for detecting the addition of similar or lower-cost EOs. Moreover, some EOs have unique or unusual marker compounds that can be used as reliable indicators of authenticity. For example: *cis*-anethole/anisyl alcohol in *Illicium verum*, (−)-lavandulol/(−)-lavandulyl acetate in *Lavandula augustifolia*, and menthofuran in *Mentha* x *piperita*, among others [66]. In addition, the enantiomeric excess of (±)-α-pinene has shown to be very useful in identifying differences between primary and commercially available EOs of *Pinus sylvestris*, revealing potential mislabeling, incorrect herbal sources, or adulteration [67]. Furthermore, chamomile (*Chamomilla recutita*) EO is frequently adulterated with oils from other plants that contain α-bisabolol, its primary compound. Although α-bisabolol occurs in four stereoisomeric forms, only one of these ((–)-α-bisabolol) is present in authentic chamomile oil [68].

Adulteration with synthetic compounds represents another significant concern. In these cases GC–isotope ratio mass spectrometry (GC–IRMS) is particularly useful to distinguish natural from synthetic compounds based on isotopic signatures. For example, synthetic (*E*)-anethole added to fennel, star anise, or anise EOs can be identified through distinct ^13^C/^12^C ratios [69]. Similarly, the addition of racemic linalool and linalyl acetate to bergamot (*Citrus bergamia*) EO can be detected through enantioselective analysis, as natural oils predominantly contain the (R)-enantiomers of these compounds [70].

Additionally, vegetable oils like castor, sunflower, maize, canola, and nut oils are frequently added to increase EOs volume, as they are cheaper and generally odorless. This type of adulteration is one of the most common due to vegetable oils’ low cost and high availability, as well as the difficulty in identifying their presence by gold standard GC. Therefore, alternative techniques such as ^13^C NMR spectroscopy have been applied to assist in the identification of these adulterations [71]. Importantly, this technique is not influenced by the specific combination of EO and vegetable oil [61]. By analyzing both ^1^H and ^13^C glycerol backbone signals, which are characteristic of all vegetable oils containing triglycerides, the method successfully detected the addition of corn oil to rosemary EO in varying proportions.

A combination of multiple methods has also been explored to improve the accuracy of both EO quality assessment and adulteration detection, as each technique serves to validate and complement the others. For instance, the combined use of chiral and isotope analysis (^13^C/^12^C and D/H) for citronellal and citral played a crucial role in ensuring authenticity and detecting the presence of synthetic compounds in commercial EOs [72].

Importantly, any alteration in EO composition due to adulteration can compromise the consistency and reproducibility of their biological activity, including anticonvulsant effects. Since bioactivity is directly linked to the presence and relative abundance of specific constituents, even small compositional changes may lead to altered pharmacological responses. This highlights the need for rigorous authentication and standardization of EOs to ensure reliable and reproducible bioactivity in pharmacological and clinical research.

## 5. Anticonvulsant Mechanisms of Essential Oils and Their Constituents

Epilepsy is a chronic neurological disorder that affects around 50 million people worldwide, and with prevalence in low and middle income countries [73]. The disorder is characterized as an imbalance between inhibitory and excitatory neurotransmissions in the central nervous system (CNS) [74]. People with epilepsy present recurrent seizures, and these cause molecular changes and activation of neurodegenerative pathways related to epileptogenesis [74,75].

Certain EOs presented relevant anticonvulsant activity in experimental models. This activity involves activation of varied neurotransmission pathways and reduced neuroinflammation [76,77,78]. For the treatment of epilepsy, EOs are a promising intervention since the anticonvulsant drugs currently marketed are not fully effective, as these medications have limited efficacy, risk of serious complications, and challenges regarding treatment adherence [79].

### 5.1. Essential Oils and the GABAergic System

The GABAergic system is vital for normal brain function and is formed by cells that produce the inhibitory neurotransmitter GABA and its receptors [80]. Deregulation of this system promotes the development of neurological diseases such as schizophrenia [81], depression [82], and epilepsy [83], Table 2.

Changes in the signaling and expression of the GABAA and GABAB receptors are associated with the onset of seizures [84,85]. GABAA ionotropic receptors, depending on their conformation, allow chloride ions to enter the cell, presenting quick inhibitory effects, while the GABAB metabotropic receptors regulate potassium efflux and block calcium channels [86].

Many EOS interact with the GABAergic system and promote anticonvulsant activity. Such findings have been observed mainly with models using chemical agents, such as pentylenetetrazole (PTZ) and picrotoxin, which induce seizures in animals by blocking GABAA receptors. The EOs of *Carum carvi* L. [76], *Cinnamosma madagascariensis* Danguy [87], *Elettaria cardamomum* (L.) Maton [88] and *Piper guineense* Schum & Thonn [89], for example, are able to reduce the percentage of seizures induced by these chemical agents in animals when compared to untreated controls. *Piper guineense* Schum & Thonn contains the terpenoids *α*-pinene [90], *β*-pinene [90], linalool [91], and *β*-caryophyllene [92,93] with anticonvulsant activity already reported.

The EOs, in addition to being effective in PTZ test models, are antagonized by flumazenil administration, a competitive antagonist of the GABAA receptor at the benzodiazepine site, suggesting their strong relationships with the GABAergic system. *Satureja bachtiarica* Bunge EO prevents PTZ-induced seizure due to its effect on the GABAA receptors possibly attributed to the active compounds, such as thymol and carvacrol [94]. In the zebrafish model of seizure, *Cymbopogon citratus* (DC.) Stapf EO and its active compounds, citral and geraniol, showed antiepileptic properties with involvement of GABAA receptors and antioxidant effects in brain homogenates [95].

*Zhumeria majdae* Rech. EO (ZMEO), which the main chemical constituent is linalool, increases nitric oxide production in the CNS, which inhibits GABA transaminase, an enzyme responsible for GABA degradation, raising GABA levels sufficiently which protects against the convulsive effects of PTZ [78].

Such promising results involving EOs can be attributed to their constituents, such as *β*-caryophyllene [92,93], curcumol [96], and perillyl alcohol [97]. The monoterpene carvacryl acetate, an acetylated derivative of carvacrol, increases GABA concentrations in the hippocampus, as well as Na^+^, K^+^-ATPase, and *δ*-aminolevulinic dehydratase antioxidant activity [98].

Electroencephalographic records obtained from PTZ tests are another important tool to analyze involvements in the GABAergic system. The EO of *Pimpinella anisum* L., known as fennel oil, prolongs latency to seizure attacks and reduces the frequency, amplitude, and duration of explosive epileptiform discharges induced by intraperitoneal PTZ injection [99]. Terpinen-4-ol monoterpene reduces the number of electroencephalogram peaks as well attenuating the typical behavioral crises caused by PTZ. The monoterpene also reduces seizures caused by mercaptopropionic acid, (which itself lowers GABA production by inhibiting glutamic acid decarboxylation). These results suggest that the GABAergic system (whether directly or indirectly) is affected by the activity of terpinen-4-ol [100].

When studying GABAA receptor modulation (by EO constituents) electrophysiological studies are essential. It was observed that the monoterpenes linalool and carvacrol potentiate GABAA receptor currents [91]. Linalool metabolites present lower allosteric potential at GABAA receptors than the linalool molecule itself [101]. Linalool oxide, synthesized by natural oxidation of linalool, also presents involvement with the GABAergic system in the PTZ test, further suggesting the monoterpenes’ GABAergic structure-activity relationships [102]. Similar results have been observed with tetrahydrolinalool, a monoterpene produced during the metabolism of linalool, as evidenced in electrophysiological and molecular docking studies [103].

Research with nerve terminals isolated from the cerebral cortex of mice has revealed that (+)-Dehydrofukinone (DHF), isolated from the EO of *Nectandra grandiflora* Ness, induces sustained hyperpolarization (dependent on GABA), and decreases KCl evoked calcium mobilization, suggesting that DHF’s anticonvulsant properties involve neuronal GABAergic inhibition [104].

GABAergic interneurons can be modulated through the A2A adenosine receptor, which, when activated, increases GABA release in the hippocampus [105]. D-limonene exerts its anticonvulsant effect due to agonist activity at the A2A adenosine receptor and consequently regulates GABAergic neurotransmission [106].

### 5.2. Essential Oils and the Cholinergic System

The cholinergic system is formed by neurons that produce acetylcholine, a neurotransmitter involved in cognitive functions such as learning and memory [107]. Dysfunctions in the cholinergic system, when related to the muscarinic and nicotinic receptors present in the hippocampus and cerebral cortex, often cause epilepsy [107,108]. Studies with high doses of pilocarpine, a cholinergic agonist, increase acetylcholine levels in the hippocampus, which are responsible for inducing an epilepticus status characterized by subentrant epileptic seizures and cholinergic symptoms such as tremors, salivation, and diarrhea [109].

Interactions between the cholinergic system and EOs have been evidenced for *Cymbopogon citratus* (DC) Stapf EO which was found to increase the time to onset of seizures and extend latency to death in mice when induced by pilocarpine [109]. Similar results were obtained with the monoterpene epoxy-carvone, where there was a reduction in parameters related to cholinergic receptor activation and an increase in latency to seizure onset [110]. Acetylcholinesterase is responsible for hydrolyzing acetylcholine, and preventing cholinergic over-stimulation (occurring in the initial phase of seizures) [111].

Alpha-asarone, although it prolongs onset times in nicotine-induced seizures in mice, it does not antagonize nicotinic receptors [112].

### 5.3. Essential Oils and the Glycinergic System

Glycine is a neurotransmitter mainly released in the brain stem and spinal cord. At low concentrations, glycine acts as an *N*-methyl-*D*-aspartate (NMDA) excitatory receptor agonist, resulting in seizures. However, higher concentrations can suppress epileptiform activity [113]. This inhibitory effect occurs through activation of glycine receptors that allow the influx of chloride ions into the neuron, hyperpolarizing the cell [114].

Strychnine is an alkaloid extracted from the plant *Strychnos nux-vomica*. It causes seizures due to competitive post-synaptic inhibition of glycine receptors. This results in involuntary muscle contractions and seizures, which can lead to death [115]. In an experimental model with mice, *Dennettia tripetala* G. Baker EO did not protect against strychnine-induced seizures, but reduced the mortality caused by the alkaloid, suggesting a possible glycine pathway for interaction in the reported activity [116]. According to Sancheti et al. [117], thymol presents anticonvulsant activity without interacting with glycine receptors.

### 5.4. Essential Oils and the Glutamatergic System

Glutamate is an excitatory neurotransmitter found in the mammalian brain and is responsible for essential brain functions, such as learning and memory [118]. The actions of glutamate are mediated by both ionotropic (NMDA, AMPA, and Kainate) and metabotropic receptors. In convulsive crises, increases in extracellular glutamate contribute to excitotoxicity, as do changes in neuronal and glial receptor expression that favor epileptogenesis [119].

Linalool, a major compound of *Ocimum basilicum* L. oil, presented anticonvulsant activity via modulation of the glutamatergic system in both in vivo and in vitro studies, revealing direct interactions with NMDA receptors [120,121]. Linalool may also be responsible for reducing the number of seizures and increasing the latency time of the first seizure in animals treated with *Mentha piperita* L. [122]. In electrophysiological studies by Wang et al. (2023), it was observed that (+)-borneol enantiomer, a monoterpene compound, has anti-seizure potency in the long term via decreasing the glutamatergic synaptic transmission [123].

### 5.5. Essential Oils and Seizure-Induced Oxidative Stress and Inflammation

Researchers around the world have been dedicated to understanding the association between seizures and oxidative stress [124]. The brain is highly dependent on oxygen to perform its functions, and is susceptible to oxidative stress. When exacerbated, oxidative stress can induce neuronal apoptosis and epilepsy [12]. The administration of convulsive doses of PTZ and pilocarpine induces neuronal damage, leading to an increase in oxidative stress indicators, and decreased antioxidant enzyme activity [125,126,127].

In this context, certain EOs and their constituents have become promising oxidative damage limiters due to their antioxidant potential. For example, the EO extracted from fresh aerial parts of *Ducrosia anethifolia* Boiss and its constituent α-pinene were found to reduce the duration of PTZ induced seizures, and their antioxidant properties were revealed in the temporal lobe of rats [128]. This antioxidant potential was also observed in the EO from *Melissa officinalis* L. (Lemon balm) accompanied by an improvement in the depressive-like behavior, cognitive deficits, and neuronal cell loss in the hippocampus of PTZ-kindled rats [109]. This anticonvulsant activity can be attributed to geranial and neral monoterpenes (25.57 and 19.54%, respectively) present in other EOs with the same pharmacological effects [128].

As expected, the compounds derived from EOs have robust neuroprotective properties in various degenerative models. In this sense, gamma-decalactone monoterpene, while not being able to protect against seizures induced in the pilocarpine model, presents a neuroprotective effect by reducing both oxidative stress and DNA damage, and without inducing gene mutations [127]. In a study by Felipe et al. (2019) [90], the isomer *β*-pinene (different from *α*-pinene) was effective during PTZ tests. Treatment with *β*-pinene, in an equimolar mixture of its two structural isomers, significantly reduced the level of reactive nitrogen species originating in NO metabolism. *β*-Pinene also reduces the increasing concentrations of dopamine and norepinephrine generally caused in PTZ induced seizures [90]. α- and *β*-Pinene isomers contribute to the positive responses induced by the administration of the EO of *Ferula gummosa* Boiss in mice [129].

Oxidative stress is directly linked to the inflammation present during epileptogenesis. This neuroinflammation is closely related to elevated levels of pro-inflammatory cytokines such as interleukin-1 (IL-1*β*), tumor necrotic factor-alpha (TNF-*α*), and interleukin-6 (IL-6), as well as to increased protein expression of serum levels of caspase-3, BAX, and BH3 [130]. In this context, the stereoisomer (+)-*cis*-epoxy-carvone, a monoterpene of natural origin, decreases pro-inflammatory levels of the cytokines IL- 1*β*, IL–6, and TNF*α*, and further, was found to present neural protection in an epileptogenesis model in mice [131]. In a kindling model, the borneol monoterpene suppressed seizures by attenuating both oxidative stress and neuroinflammation by reducing the levels of glial fibrillary acidic protein (GFAP), a marker of brain damage [132]. Alpha-asarone also presents an anti-inflammatory profile; it inhibits NF-*κ*B pathway activation in the microglia, making it a future option for the treatment of epilepsy [133].

Moreover, the monoterpene (*1S*)-(−)-verbenone presents anticonvulsant activity through increased expression of brain-derived neurotrophic factor (BDNF), responsible for neuronal survival and growth, and through negative regulation of COX-2 and c-fos mRNA [134]. Similar results were also observed with citral monoterpene that was able to increase latency until the first seizure and reduce neuronal death in the hippocampus, and expression of the BDNF, TNF-*α*, IL-*6*, IL-1*β*, and NF-*κ*B genes [135].

The monoterpene carvacrol’s anticonvulsant effect is also attributed to the reduction of COX-2 levels in the hippocampus [136]. In addition, carvacrol, by blocking transient receptor potential cation subfamily M7 (TRPM7) channels, prevents neuronal death in the hippocampus, as well as memory deficits that occur after experimentally induced status epilepticus [137].

Nuclear factor erythroid 2-related factor 2 (Nrf2, or NFE2L2) participates in the body’s natural cellular defense system by activation of antioxidant gene expression, thereby preventing neuronal damage induced by ROS. In addition, activated Nrf2 exerts an anti-inflammatory effect by crosstalk with NF-*κ*B [138]. Against this background, monoterpene carveol reversed the seizures induced by PTZ via activation of Nrf2 and, consequently, mitigated inflammatory insults through multiple pathways [139].

**Table 2:** Neurotransmission pathways involved in the anticonvulsant activity of essential oils.

Mechanism of Action	Essential Oils	Chemical Composition of the Essential Oil	Reference
GABAergic system modulation	*Annona vepretorum* Mart. (Annonaceae)	bicyclogermacrene (39%), spathulenol (14%), α-phellandrene (11.5%), (*E*)-β-ocimene (8.6%), *o*-cymene (6%), α-pinene (6%) and germacrene D (5.5%)	[140]
	*Carum carvi* L. (Apiaceae)	Not provided	[76]
	*Cinnamosma madagascariensis* Danguy (Canellaceae)	linalool (30.1%), limonene (12.0%), myrcene (8.9%), and *α*-pinene (8.4%)	[87]
	*Citrus aurantium* L. blossoms (Rutaceae)	linalool (28.5%), linalyl acetate (19.6%), nerolidol (9.1%) *E*,*E*-farnesol (9.1%), α-terpineol (4.9%) and limonene (4.6%)	[141]
	*Cymbopogon citratus* (DC.) Stapf (Poaceae)	Citral and geraniol	[95]
	*Dennettia tripetala* G. Baker (Annonaceae)	1-nitro-2-phenylethane (80%), β-eudesmol and nerolidol (4%), 1-linalool (11%), β-caryophyllene and β-humulene	[116]
	*Dorema ammoniacum* gum (Apiaceae)	Not provided	[142]
	*Elettaria cardamomum* (L.) Maton (Zingiberaceae)	1,8-cineole (45.6%), α-terpinyl acetate (33.7%), sabinene (3.8%), 4-terpinen-4-ol (2.4%), and myrcene (2.2%)	[88]
	*Pimpinella anisum* L. (Apiaceae)	*trans*-anethole (89.1%), estragol (3.6%), linalool (1.1%), *α*-terpineol (0.2%) and *cis*-anethole (0.2%)	[99]
	*Piper guineense* Schum & Thonn (Piperaceae)	β-sesquiphellandrene (20.9%), linalool (6.1%), limonene (5.8%), Z-β-bisabolene (5.4%) and α-pinene (5.3%)	[89]
	*Ocimum basilicum* Linn (Lamiaceae)	oxygenated monoterpenes (34.67%), sesquiterpenes (0.49%), oxygenated sesquiterpenes (1.13%), and phenylpropanoids (63.72%)	[16]
	*Smyrnium cordifolium* boiss (Apiaceae)	curzerene (65.26%), δ-Cadinene (14.39%) and γ-elemene (5.15%)	[143]
	*Zhumeria majdae* Rech. (Lamiaceae)	linalool (61.4%) followed by camphor (27.5%). Other major components found include *cis*-linalool oxide (1.11%), limonene (0.98%), and geraniol (0.9%)	[78]
via cholinergic system	*Cymbopogon citratus* (DC) Stapf (Poaceae)	geranial (45.4%), neral (37.4%) and myrcene (14.4%)	[109]
Involvement of glycinergic system	*Dennettia tripetala* G. Baker (Annonaceae)	1-nitro-2-phenylethane (80%), β-eudesmol and nerolidol (4%), 1-linalool (11%), β-caryophyllene, and β-humulene	[116]
Involvement of glutamatergic system	*Mentha piperita* L. (Lamiaceae)	Not provided	[122]
	*Ocimum basilicum* Linn (Lamiaceae)	oxygenated monoterpenes (34.67%), sesquiterpenes (0.49%), oxygenated sesquiterpenes (1.13%), and phenylpropanoids (63.72%)	[16,120,121]
via oxidative stress and neuroinflammation	*Ducrosia anethifolia* Boiss (Apiaceae)	α-Pinene (11.6%), terpinolene (3.2%) and (*Z*)-β-ocimene (2.8%)	[77]
	*Ferula gummosa* Boiss (Apiaceae)	β-pinene (35.9%), sabinene (28.4%), α-pinene (13.32%), and ρ-cymene (5.11%)	[129]
	*Melissa officinalis* L. (Lamiaceae)	Geranial (25.6%), neral (19.5%), caryophyllene oxide (13.2%) and (*E*)-caryophyllene (11.6%)	[128]

Interestingly, the sesquiterpene *β*-caryophyllene has shown promise in pharmacological studies associated with Dravet Syndrome (DS) for being active against behavioral comorbidities associated with the disease, but also reduced glial activity [144]. The association between β-caryophyllene and cannabidiol potentiated the beneficial effects in mice with DS, besides reducing several inflammation-related markers [145]. DS is caused by a genetic mutation of the sodium channel nav1.1, which results in pediatric febrile seizures [146].

## 6. Conclusions

Essential oils exhibit anticonvulsant activity through multifaceted mechanisms involving the modulation of key neurotransmitter systems, namely GABAergic, cholinergic, glycinergic, and glutamatergic pathways, as well as the regulation of oxidative stress and neuro-inflammatory processes. These combined actions highlight their potential as promising candidates for seizure control. Nevertheless, their therapeutic application remains limited by factors such as chemical variability, lack of standardization, insufficient mechanistic understanding, and the lack of clinical evidence.

Importantly, the pharmacological effects of EOs are closely linked to their chemical composition, which is strongly influenced by extraction methods. Therefore, accurate characterization is essential to identify the bioactive constituents responsible for these effects. In parallel, rigorous quality assessment in accordance with internationally recognized standards, including those of the European Pharmacopoeia and the International Organization for Standardization (ISO), is necessary to ensure reliability, reproducibility, and the absence of adulteration.

## Figures and Tables

**Table 1:** Common extracts obtained from essential oils.

Name	Definition According to ISO 9235:2013 Standard
Concrete	Extract obtained from a fresh natural raw material by extraction with one or several solvents.
Resinoid	Extract obtained from a dry plant natural raw material by extraction with one or several solvents.
Pomade	Perfumed fat obtained from a flower, either by “cold enfleurage” (diffusion in particular of the odoriferous compounds of the flower in the fat), or by “hot enfleurage” (digestion or immersion of the flower in the melted fat).
Absolute	Product obtained by extraction with ethanol from a concrete, a floral pomade, a resinoid, or a supercritical fluid extract.
Supercritical fluid extract	An extract obtained by treating a natural raw material in a supercritical fluid, followed by a separation by expansion.

## Data Availability

Data available on request from the author.
